# Highly Efficient Discovery of 3D Mechanical Metamaterials via Monte Carlo Tree Search

**DOI:** 10.1002/advs.202513771

**Published:** 2025-09-23

**Authors:** Jiamu Liu, Bo Peng, Weiyun Xu, Ye Wei, Peng Wen

**Affiliations:** ^1^ State Key Laboratory of Clean and Efficient Turbomachinery Power Equipment, Department of Mechanical Engineering Tsinghua University Beijing 100084 China; ^2^ Department of Civil and Environmental Engineering University of Illinois Urbana‐Champaign Urbana IL 61801 USA; ^3^ Department of Data Science City University of Hong Kong Hong Kong China

**Keywords:** active learning, machine learning, mechanical metamaterials, Monte Carlo tree search

## Abstract

Machine learning (ML) has surpassed traditional intuition‐driven trial‐and‐error approaches in metamaterial design by employing efficient inverse pipelines based on structure–property mapping. However, three critical challenges impede the applications of ML when extending the geometry from 2D to 3D: exponentially increasing design space dimensionality, scarce high‐quality training data, and excessive computational demands. To address these problems, Monte Carlo Tree Search‐Active Learning (MCTS‐AL), an active learning framework integrating Monte Carlo Tree Search (MCTS), convolutional neural networks (CNNs), and finite element method (FEM) to efficiently explore high‐performance 3D mechanical metamaterials using only 100 initial samples within a vast design space (≈7^27^ possibilities), is proposed. Demonstrated on triply periodic minimal surface (TPMS) metamaterials for stiffness and strength optimization, MCTS‐AL achieves 30% higher stiffness than uniform designs, an enhancement of strength of more than 20% compared with benchmark active learning methods (e.g., Bayesian Optimization, BO), and fewer iterations until convergence. T‐distributed Stochastic Neighbor Embedding (T‐SNE) clustering confirms that the superior performance stems from a comprehensive understanding of the design space and diverse sampling, with optimized structures forming distinct and various clusters. This work establishes a scalable, data‐efficient strategy for high‐dimensional mechanical metamaterial design and is expected to be applied in other scenarios demanding optimal solution exploration.

## Graphical abstract

Machine learning (ML), as a data‐driven method, has revolutionized metamaterial design, surpassing traditional intuition‐driven trial‐and‐error methods in both efficiency and performance. Here, MCTS‐AL, an active learning framework integrating finite element simulation (FEM), convolutional neural networks (CNNs), and Monte Carlo Tree Search (MCTS), demonstrating superior optimization efficacy compared to existing approaches, is presented.

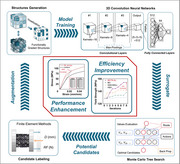

## 1 Introduction

Metamaterials, also referred to as architected materials, can exhibit unprecedented properties rarely found in conventional materials through the rational design of their building blocks.^[^ ^1^, ^2^, ^3^ ^]^ Mechanical metamaterials offer exceptional programmability and can achieve unique, counterintuitive mechanical properties and, most importantly, extraordinary mechanical properties like excellent stiffnesses^[^ ^4^, ^5^, ^6^, ^7^, ^8^ ^]^ and strengths.^[^ ^1^, ^4^, ^5^, ^9^, ^10^, ^11^ ^]^ These properties have enabled diverse applications, particularly in aerospace and defense coatings.^[^ ^12^, ^13^, ^14^ ^]^ Despite their advantages, designing mechanical metamaterials remains challenging, as their microstructure must be meticulously optimized to achieve superior performance. Traditional design methods typically follow a forward‐design approach including from conceptualization to validation, which heavily relies on the expertise of designer and involves time‐consuming trial‐and‐error iterations.^[^ ^15^, ^16^, ^17^ ^]^

Machine learning (ML) algorithms can autonomously extract key features from provided data and continuously improve their performance through experience, playing a significant role in performing classification, regression, clustering, and dimensionality reduction tasks.^[^ ^18^, ^19^, ^20^ ^]^ For instance, Wang et al. integrated machine learning with physical equations to efficiently and accurately predict the lattice thermal conductivity of crystals.^[^ ^21^ ^]^ In a similar vein, Zhu et al. utilized convolutional neural networks (CNNs) to predict the thermal conductance of complex network structures.^[^ ^22^ ^]^ Meanwhile, Wang et al. employed Kolmogorov–Arnold Networks (KANs) to model the Poisson's ratio of elastic networks, deriving an interpretable mathematical expression in the process.^[^ ^23^ ^]^ Through inverse design, a widely adopted technique using desired properties as inputs to directly generate optimized structures, offering a promising paradigm for metamaterial design.^[^ ^16^, ^24^, ^25^, ^26^, ^27^ ^]^ Inverse design typically requires a generative model that needs to be trained on an extensive dataset, which is readily accessible for 2D and truss metamaterials due to their computational simplicity in finite element simulations. However, 3D mechanical metamaterials, especially those with complex curved geometries and specialized functions, face significant challenges in terms of ML design, because these structures demand substantially more finite elements to accurately model curved surfaces. For instance, a triply periodic minimal surface (TPMS) structure (9 mm × 9 mm × 9 mm) meshed at 0.08 mm resolution contains ≈13 00 000 elements, almost several times as many as 2D structures with similar porosity. This leads to prohibitive simulation times (several hours or even several days per simulation for some specific mechanical properties) and sparse datasets that limit inverse design model training.

Therefore, recent advances in active learning (AL), which integrates ML with expert knowledge from simulations or experiments, have emerged as a promising solution for sparse‐data optimization.^[^ ^28^ ^]^ For instance, Bayesian optimization (BO) has been employed to address such data scarcity, demonstrating success in optimizing the toughness of mechanical metamaterials.^[^ ^29^ ^]^ Yet, employing Gaussian process regression (GPR) as a surrogate model, BO fails to capture complex relationships between high‐dimensional features while simultaneously demanding substantial computational resources, thereby limiting its applicability. The integration of hybridization into Evolutionary Algorithms (HEAs) has the potential to enhance performance—for instance, accelerating convergence and improving solution quality through systematic recombination.^[^ ^30^ ^]^ However, the homogenization of offspring restricts diversity in the population and ultimately hinders the algorithm's ability to explore the design space effectively, causing diminishing marginal returns and limiting further improvements in solution optimality. A framework, Generative Architecture Design – Multi‐Objective Active Learning Loop (GAD‐MALL), was proposed to address the problem, which leverages generative models to efficiently sample in the relatively high‐dimensional design space, and achieved success in optimizing the modulus and strength of a 27D metamaterial.^[^ ^31^ ^]^ However, generative models suffer from two critical limitations: substantial data required for training and computational inefficiency, which impede their broader application. Tree search methods, notably Monte Carlo Tree Search (MCTS), have emerged as a promising solution to address these challenges.

The MCTS algorithm comprises four sequential phases: selection, expansion, simulation, and backpropagation. In a selection step, MCTS explores new states shifted from the initial state (which are denoted by the leaf nodes and root node, respectively), and uses Monte Carlo rollouts to estimate the value of each node, where simulation steps are taken to reach the end state (generally a win or loss in a single game).^[^ ^32^, ^33^ ^]^ According to the results of the rollouts, both values and visitations of the nodes in a sequence are updated through backpropagation. Finally, nodes are selected according to the Upper Confidence Bound (UCB) criterion, which balances exploration and exploitation by maximizing:

(1) $$UCB=\frac{V_{i}}{N_{i}}+c\sqrt{\frac{lnN_{p}}{N_{i}}}$$

where *V_i_* is the value, *N_i_* and *N_p_* represents the visits of the node and its parent, and c is an exploration constant.

MCTS excels in high‐dimensional optimization by dynamically balancing exploration and exploitation, allowing node visit statistics to avoid local minima. Its success in complex domains like game theory (e.g., AlphaGo) suggests strong potential to overcome the “curse of dimensionality.”^[^ ^33^ ^]^ However, despite the effectiveness of MCTS in game contexts, its applications to tackle engineering problems are still fairly lacking in development.^[^ ^34^ ^]^ Traditional MCTS‐based methods are primarily employed to identify winning strategies through sequential state transitions, which differ significantly from performance optimization scenarios in mechanical metamaterial design.

**Figure 1:**
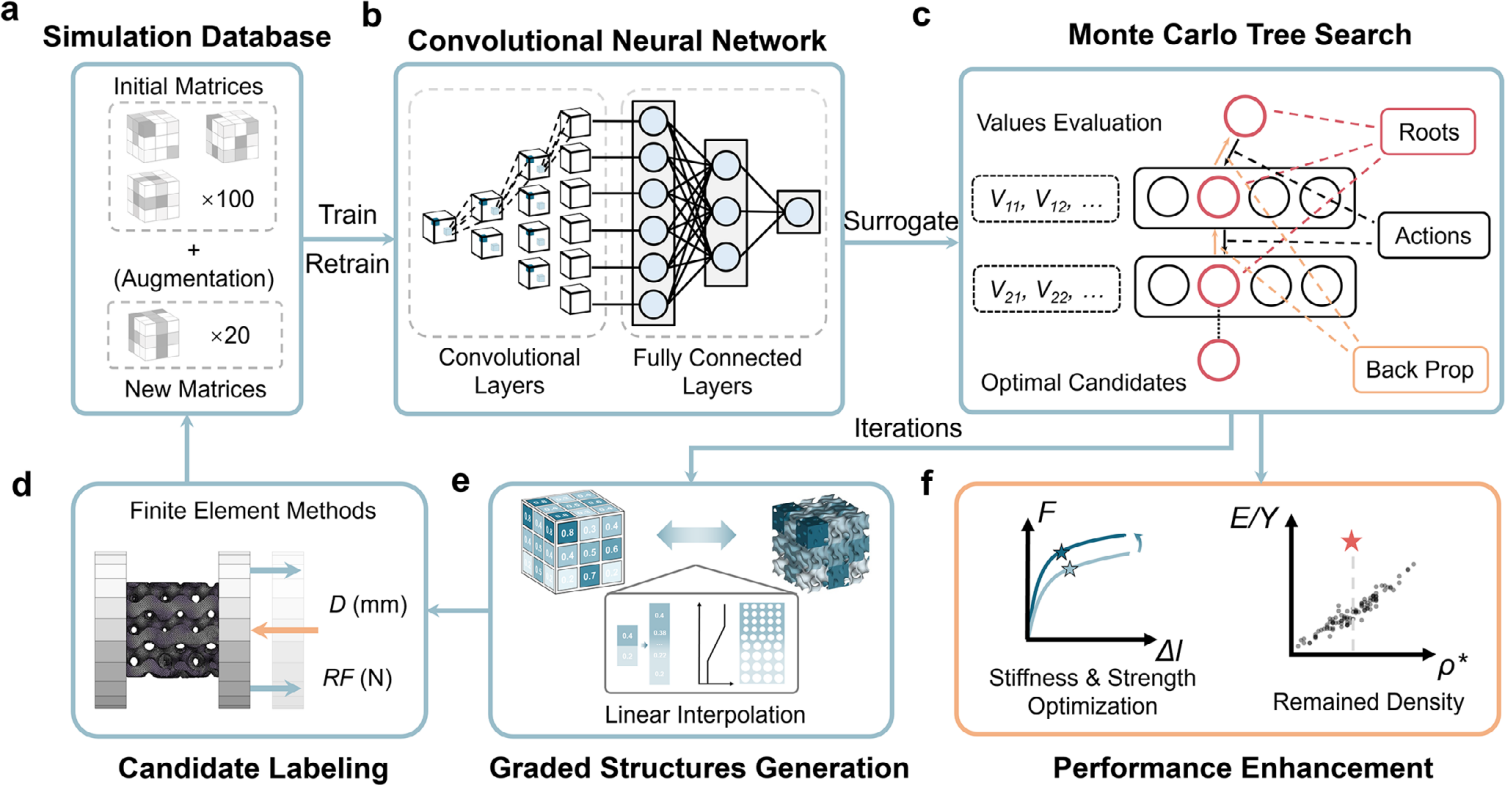
Workflow of the MCTS‐AL (Monte Carlo Tree Search – Active Learning). a) Simulation database: at the beginning of each iteration, the database is augmented with the labeled data obtained in the previous iteration. b) CNN model: convolutional layers with input size of (60, 60, 60) and fully connected layers are included. c) Structure of Monte Carlo Tree Search: new roots are selected according to the values predicted by the CNN model trained ahead. d) Labeling with FEM. e) Generating graded structures according to the volume fraction matrices, where linear interpolation is used to make the transition region smoother. f) Target of performance enhancement.

In this work, we adapted the MCTS framework, using machine learning performance predictions instead of conventional win‐condition evaluations as the value criterion for nodes. These predictions assessed the quality of individual design configurations, leading to the development of Monte Carlo Tree Search‐Active Learning (MCTS‐AL), a novel AL framework that integrates MCTS with finite element method (FEM) and convolutional neural networks (CNNs) for efficient optimization of 3D mechanical metamaterials. As a case study, we employed the Gyroid structure, a member of TPMS family, to demonstrate the framework's capabilities. In addition to the advantages of conventional TMPSs, the helical surface structure of the gyroid unit makes the force distribution more uniform, leading to its excellent mechanical properties. As illustrated in **Figure** **1**, the workflow begins with a small initial dataset (100 samples) to train an ensemble of five CNNs. MCTS then explores the design space, with CNN predictions evaluating candidate structures. The top 20 candidates undergo finite element simulation, and the results augment the training set for iterative refinement.

We validated MCTS‐AL on two critical tasks: 1) to maximize specific stiffness and 2) to maximize specific strength while maintaining the volume fractions. For stiffness optimization, MCTS‐AL identified structures achieving >30% improvement in elastic modulus compared to uniform Gyroid designs with the same volume fractions, while also outperforming conventional reference structures. For the strength optimization task, MCTS‐AL was evaluated against three benchmark algorithms—HEA, BO, and GAD‐MALL—under a strict computational time constraint of three days. Within this limited timeframe, MCTS‐AL outperformed all three methods in both optimization performance and generalization capability. The algorithm successfully generated magnesium (Mg) alloy metamaterials exhibiting strength enhancements greater than 20%, achieving convergence in only 7 iterations, with each iteration requiring ≈6 h of computation. These results highlight MCTS‐AL's potential as a scalable, high‐performance framework for advancing 3D mechanical metamaterial design.

## 2 Results and Discussion

### 2.1 Specific Stiffness Optimization with MCTS‐AL

The MCTS‐AL framework was applied to optimize specific stiffness, aiming to enhance elastic modulus while maintaining a near‐constant volume fraction. The target volume fraction was set to 0.5, with an allowable deviation of ± 5% (i.e., 0.475–0.525). By fixing the volume fraction, we investigated how mass distribution influences mechanical performance, isolating structural topology as the primary variable.

#### 2.1.1 Surrogate Model Analysis

**Figure 2:**
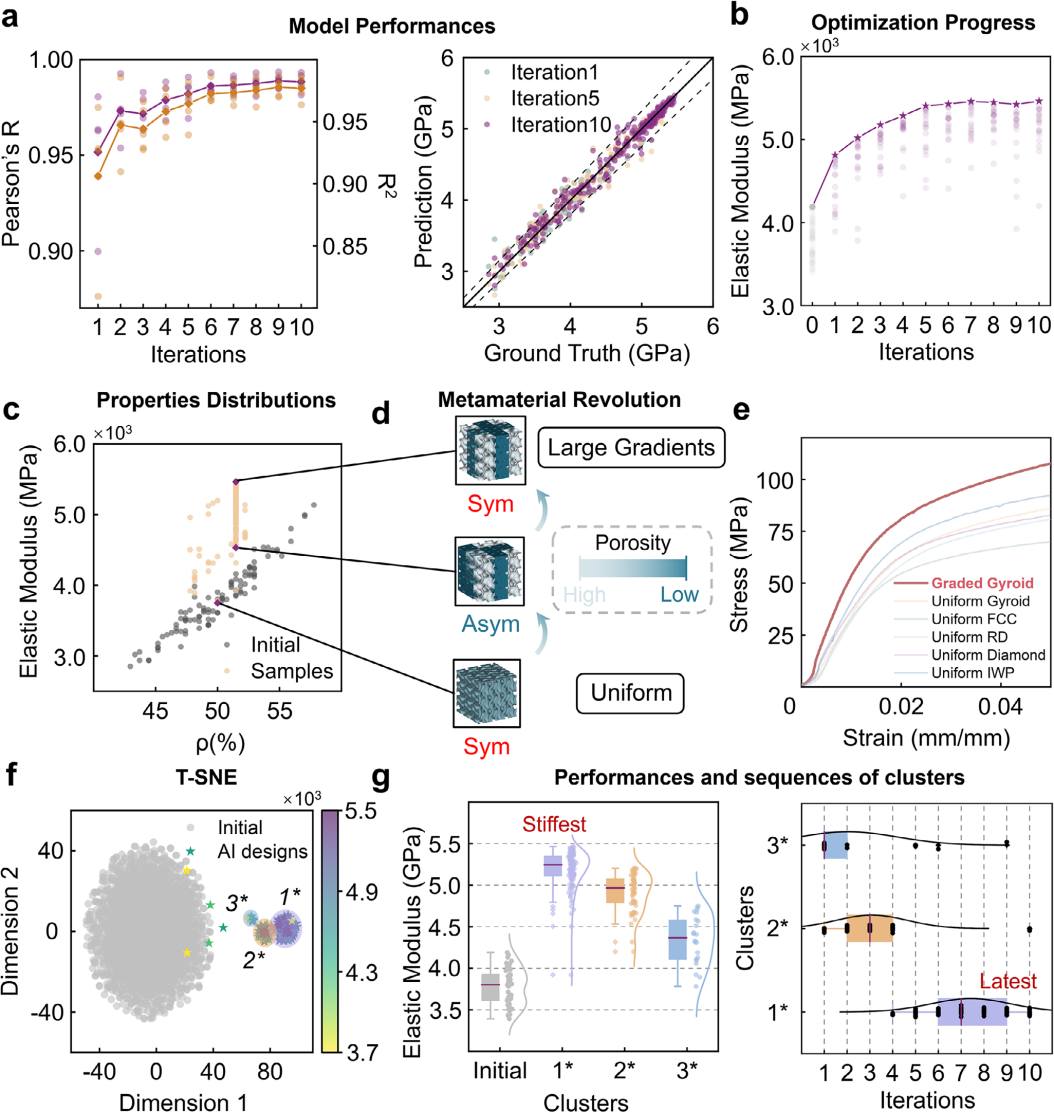
Specific stiffness optimization using MCTS‐AL. a) Performance of CNN models: Pearson's R (purple) and coefficient of determination R^2^ (yellow) across optimization iterations (left), and prediction accuracy for 1st, 5th, and 10th iterations (right). The translucent circular points represent the performance of each individual model, while the diamond‐shaped markers indicate the corresponding average value across all models. b) Optimization progress over successive iterations. c) Distribution of specific stiffness values among sampled structures. The translucent circles denote the elastic modulus values of all 20 candidate structures selected in a single iteration, and the five‐pointed star markers represent the maximum value among them in each iteration. d) Evolution of sampled metamaterial structures during optimization. Regions with light coloration indicate high porosity, while areas of dark coloration correspond to low porosity. e) Compression test results comparing optimized graded and uniform Gyroid TPMS structures with several conventional designs. f) Dimensionality reduction and clustering of all structures designed by MCTS‐AL. g) Performance comparison (left) and iteration‐wise distribution (right) of the three identified clusters.

The predictive performances of the surrogate models were systematically evaluated. As illustrated in the left panel of **Figure** **2**a, all models (except one outlier in the first iteration) achieved high accuracy, with Pearson's correlation coefficient (R) exceeding 0.95. Furthermore, the CNN models exhibited a consistent upward trajectory in prediction accuracy as optimization progressed. The combination of high baseline accuracy and iterative refinement established a robust foundation for identifying high‐performance candidates.

During initial iterations, model accuracies varied between 0.9 and 0.98, reflecting lower average performance and higher variance, which is a consequence of the limited initial dataset (number of samples = 100). As optimization advanced, accuracy stabilized within a narrow range of 0.98–0.99, accompanied by reduced variance. To contextualize these improvements, Figure 2a (right panel) compares FEM ground truths with CNN predictions across iterations. There are two key drivers that lead to the accuracy gains. First is dataset expansion. Progressive growth of training and validation sets provided richer input‐output relationships for feature learning. Property distribution shift is the other. Early iterations featured samples with elastic moduli of 25–50 MPa, while later iterations prioritized designs exceeding 50 MPa, aligning with the models’ optimized predictive range. These dynamics collectively enhanced model reliability, enabling efficient exploration of high‐performance candidates.

#### 2.1.2 Optimization Results

The optimization continued for 10 iterations, starting with an initial dataset of 100 structures, and concluding when no significant performance gains were observed over three consecutive iterations to save the computational expenses and avoid diminishing marginal returns in the following iterations. In total, 180 effective structures were sampled (20 were excluded due to a huge difference), with the entire process requiring 3–4 days to complete. Each iteration took ≈6 h to converge. As shown in Figure 2b, the elastic modulus improved by 30% (from 4189.7 to 5464.7 MPa) after 10 iterations, aligning closely with the progressive accuracy gains of the surrogate models. Notably, candidates within individual iterations exhibited significant property variability, a consequence of selection criteria prioritizing comprehensive performance and sample diversity (see Experimental Section ‘Query Strategy’).

The relationship between volume percentage (*ρ*) and elastic modulus is visualized in Figure 2c. Initial samples clustered within a narrow band, whereas MCTS‐AL‐derived candidates predominantly occupied regions above this band. A striking vertical cluster at *ρ* = 51.48% emerged, representing a dominant structural family. This phenomenon reflects the tree policy (see Experimental Section ‘Query Strategy’), which balances mechanical performance (guiding root node selection) with volume fraction stability between root and leaf nodes, thereby constraining most optimizations to a narrow *ρ* range.

We then discussed the further insights into the main cluster. Key structural transitions during optimization are illustrated in Figure 2d. Evolution occurred in two phases: 1) nonhomogenization of porosity distribution and 2) unit‐cell volume fraction refinement. To characterize the design space, we applied T‐distributed Stochastic Neighbor Embedding (t‐SNE) to project high‐dimensional structural data into a 2D latent space (Figure 2f). The initial analysis included 280 designs (100 initial structures + 180 optimized AI‐generated candidates), which proved insufficient to comprehensively characterize the design space. To address this limitation, we expanded the dataset to 2000 structures using Latin Hypercube Sampling (LHS, see Experimental Section ‘Structures Generation Strategy’), which ensures uniform spatial coverage across the entire design space while simultaneously providing a stratified representation of all variable margins.

The t‐SNE projection revealed three distinct clusters (Figure 2f). Cluster 1, which consisted of samples explored mainly in the later iterations and associated with the highest‐performing designs, exhibited superior elastic modulus values and demonstrated significant gradients (Figure 2g, left). Clusters 2 and 3, primarily populated by early iteration samples, demonstrated progressively lower median performances and are more uniform compared with Cluster 1. Temporal analysis of sampling sequences (Figure 2g, right) confirmed that Cluster 1 dominance emerged in later iterations, reflecting the optimization framework's progressive refinement toward high‐performance regions. In conclusion, Cluster 1 possesses the highest average and median elastic modulus of 5.25 GPa, predominantly comprising late‐iteration designs, while clusters 2–3, mainly discovered in early iterations, demonstrate lower performance metrics with averages of 4.91 and 4.30 Gpa, respectively. This stratification underscores the iterative efficacy of MCTS‐AL, which systematically shifts exploration toward high‐performance subspaces while maintaining diversity to avoid premature convergence.

To quantify the impact of unit‐cell volume fraction (*ρ*) distribution on overall mechanical performance of structures, we analyzed the 27‐unit‐cell matrix using a coordinate system $\delta =(\begin{array}{ccc} \delta_{x} & \delta_{y} & \delta_{z} \end{array})$, where each δ_*i*_ ε {0, 1,  2} corresponds to discrete spatial positions (Figure S1b, Supporting Information). From a global point of view, high‐performance designs (cluster 1) exhibited pronounced *ρ* gradients (more appearances of extremes like 0.2 and 0.8), diverging from the uniform or random distributions typical of the initial structures (Figure S2, Supporting Information).

Specific to each unit cell, a notable finding was the emergence of *z*‐axis consistency in optimized structures. In other words, unit cells parallel to the axis z like (δ_*x*_, δ_*y*_, 0), (δ_*x*_, δ_*y*_, 1), and (δ_*x*_, δ_*y*_, 2) demonstrated extremely similar distributions. Despite initial stochastic porosity distributions across 27 unit cells, optimized candidates exhibited uniform layering perpendicular to the z‐axis. This contradicted the initial assumptions that structural symmetry might differentiate the middle layer's influence. To validate this trend statistically, we generated two datasets: 1000 structures with randomized layer distributions and 1000 with z‐axis‐uniform layers, both at *ρ* = 0.5. CNN predictions (Figure S1a, Supporting Information) revealed that z‐axis‐uniform structures achieved higher average performance and a superior performance ceiling. Additionally, rotating layers to disrupt z‐axis consistency (Figure S1c, Supporting Information) significantly degraded the performance, confirming the critical role of axial uniformity. From a mechanical standpoint, structures lacking this uniformity are susceptible to localized failure in specific layers—even when other layers operate well below their yield strength—resulting in compromised overall mechanical performance. This phenomenon is not limited to gyroid TPMS architectures; porous metamaterials across diverse structural types exhibit similar behavior. Consequently, the deliberate incorporation of axis‐specific uniformity in graded structural design may offer significant advantages when optimizing for mechanical performance.

Then, focusing on a single z‐axis‐perpendicular layer, we categorized unit cells into corners, sides, and centers (Figure S1b, Supporting Information). For those high‐performance designs (cluster 1), the centers have volume fractions of 0.8. Of four sides, two positions (e.g., (1, 0, δ_*z*_) and (2, 1, δ_*z*_)) showed volume fractions of 0.7–0.8; others exhibited broader distributions with relatively high medians. As for corners, three positions (e.g., (0, 0, δ_*z*_), (2, 0, δ_*z*_), and (2, 2, δ_*z*_)) maintained minimal volume fractions of 0.2, with one outlier showing high volume fractions. These findings underscore a hierarchical influence: center regions exert the greatest impact on mechanical performance, followed by sides, and finally corners. Theoretically, perfect symmetry would yield a high‐volume fraction cross (with *ρ* = 0.7–0.8) centered on the layer, flanked by low‐volume fraction corners (*ρ* = 0.2). However, few AI‐generated designs matched this ideal, likely due to FEM inaccuracies (see Experimental Section ‘Labeling with FEM Method’). Despite parameter calibration, discrepancies persisted between FEM predictions and experimental validations, underscoring the sensitivity of active learning outcomes to dataset quality.

**Figure 3:**
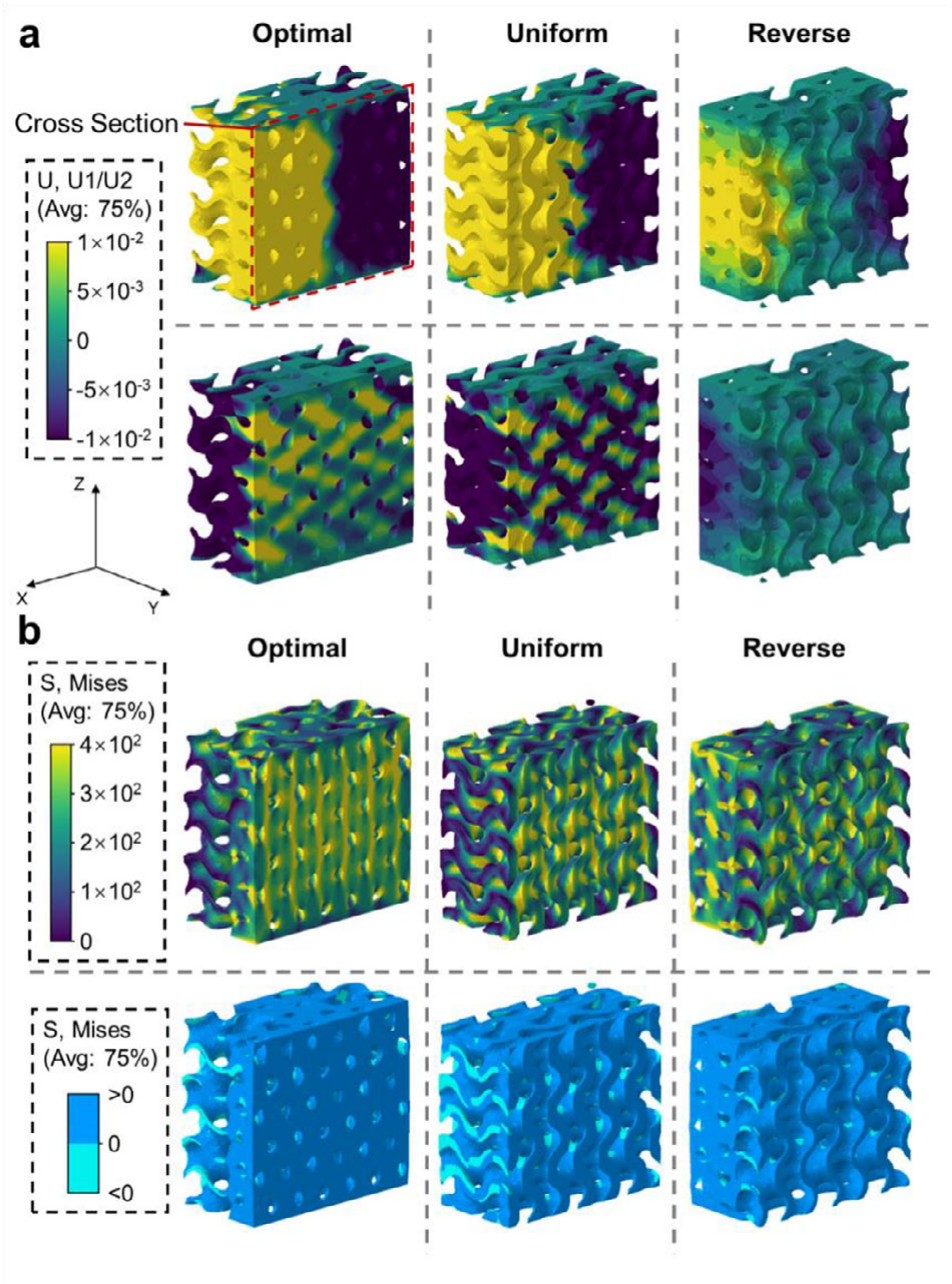
Cross‐sectional views of FEM analysis with displacement distributions a) and stress distributions b) under 0.6 mm deformation for three designs: optimized structure by MCTS‐AL (left), uniform structure (middle), and reverse structure (right).

FEM results robustly validated the superior mechanical performance of MCTS‐AL‐optimized designs. **Figure** **3** compares displacement, von Mises stress and pressure distributions across three configurations: the ML‐optimized structure, its geometric inverse (generated via a volume fraction matrix *M*_Rev_ =  1 − *M*_opt_, where *M*_opt_ is the optimized volume fraction matrix), and a uniform reference. As evidenced in Figure 3a, under certain identical axial (*z*‐directional) displacement constraints, the ML‐optimized design exhibited more uniform U1 and U2 displacement fields compared to the other structures. This uniformity suggests a prevalence of stretch‐dominated deformation mechanisms in the optimized architectures, which is a hallmark of efficient load transfer that directly correlates with enhanced stiffness characteristics according to the Gibson–Ashby theory. These observations validate the superior mechanical performance of the generated ML designs. As shown in Figure 3b, the ML design exhibited significantly higher von Mises stress magnitudes, indicating the efficient load transfer along with markedly uniform stress distribution compared to the inverse and uniform structures.

The elevated stress magnitudes and homogeneity observed in the optimized design reflect enhanced structural integrity and load‐bearing capacity. This improvement arises from the rational spatial allocation of stiffened and compliant regions within the metamaterial. In contrast, uniform designs with identical volume fractions lack such tailored stress modulation, resulting in suboptimal load distribution. These findings confirm that functionally graded architectures outperform homogeneous counterparts in specific stiffness, demonstrating the design efficacy of MCTS‐AL for 3D mechanical metamaterial.

### 2.2 Specific Strength Optimization with MCTS‐AL

**Figure 4:**
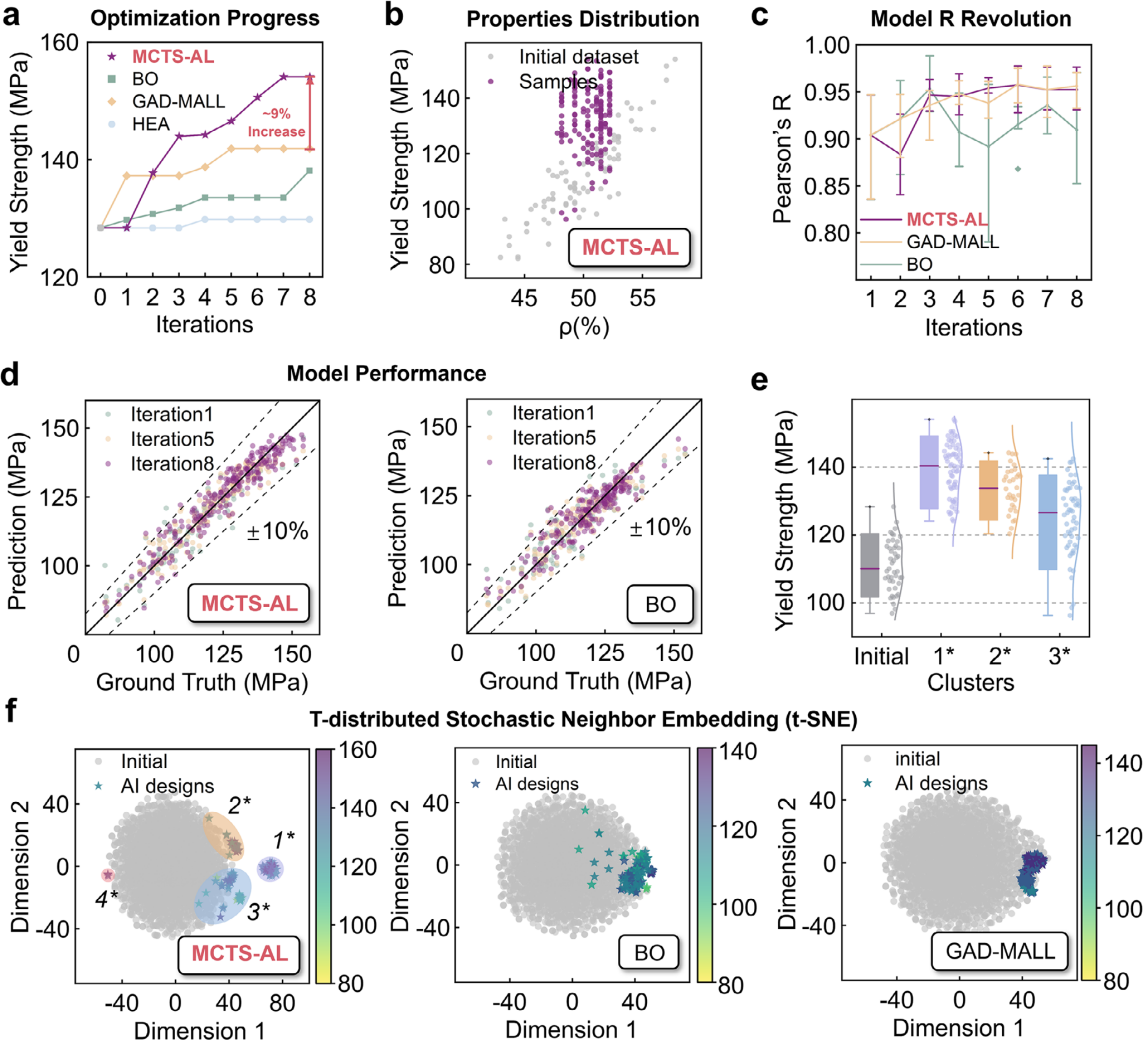
Results of specific strength optimization using three different methods: HEA, BO, GAD‐MALL, and MCTS‐AL. a) Properties of the top 20 candidate structures sampled in each optimization iteration across the three methods. b) Scatter plot showing the properties distributions between volume fraction (*ρ*) and yield strength for samples generated by MCTS‐AL. c) Performance of machine learning surrogate models throughout the optimization process for each method. d) Comparison between FEM‐evaluated ground truth and CNN‐predicted properties during model training at iterations 1, 5, and 8: MCTS‐AL (left), BO (right). e) Performances of different clusters explored by MCTS‐AL. f) Dimensionality reduction and clustering of structures designed by the three methods via T‐SNE.

While MCTS‐AL demonstrated success in optimizing specific stiffness, its performance ceiling remains an open question. To further evaluate its capabilities and robustness, we extended the framework to optimize specific strength, a more challenging task due to the nonlinearity of yielding behavior and the heightened sensitivity of yield strength to volume fraction gradients. These nonlinear relationships complicate feature extraction for ML algorithms and demand greater robustness in surrogate modeling. For comparative evaluation, MCTS‐AL was benchmarked against three alternative optimization methods: HEA, BO, and GAD‐MALL. The results, summarized in **Figure** **4**, indicate that MCTS‐AL achieved a 20% enhancement in yield strength, reaching 154.1 MPa, significantly outperforming both GAD‐MALL (10% improvement) and BO (4% improvement). Meanwhile, HEA showed negligible optimization progress and was consequently excluded from further analysis. The corresponding accuracies of the surrogate models used in each method are detailed in Figure 4c.

#### 2.2.1 Surrogate Model Analysis

CNN models in this task underperformed relative to stiffness optimization, reflecting the complexity of strength prediction. MCTS‐AL and GAD‐MALL exhibited steady accuracy gains, whereas BO suffered from oscillations and stagnation (Figure 4c). Scatter plots comparing FEM ground truths and CNN predictions (Figure 4d) revealed some critical insights. For BO, little progress has been made in the optimization, leading to property distribution constrained to the initial dataset. Training data for BO's surrogate models lacked diversity, leading to poor generalization. Local Optima occurred in the optimization by GAD‐MALL: By iteration 8, GAD‐MALL's training data clustered narrowly ≈140 MPa, yielding high localized accuracy but limited exploration. In contrast, MCTS‐AL demonstrated advantages superior to those of the two methods. MCTS‐AL maintained broad property coverage while achieving comparable accuracy, enabling balanced exploration‐exploitation. From mechanical point of view, MCTS‐AL's superiority stems from its dynamic sampling strategy, which prioritizes diverse candidates through a comprehensive Performance Criteria that integrates property targets and design‐space distance (see Experimental Section ‘Query Strategy’). Besides, unlike the generative model in GAD‐MALL, the flexibility of tree search contributes to avoiding over‐reliance on historical optima, leading to better fitness of surrogate models. This approach prevented premature convergence, allowing MCTS‐AL to identify high‐strength designs inaccessible to the rival methods.

#### 2.2.2 Optimization Results

All three methods except HEA achieved measurable improvements in yield strength, though with notable disparities in efficacy, as shown in Figure 4a. HEA exhibited a pattern of diminishing marginal returns, consistent with its performance in stiffness optimization, though its effectiveness was further reduced under the increased complexity of strength‐oriented tasks (see Figure S8, Supporting Information). This suboptimal performance was largely attributable to offspring homogenization, which substantially limited the algorithm's exploratory capacity and hindered the overall optimization outcome. BO increased yield strength by 4% (from 128.37 to 133.51 MPa). Analysis of its sampled structures (Figure 4f) revealed minimal deviation from the initial dataset, suggesting insufficient exploration of the design space and revealing its limitations in high‐dimensional optimization. GAD‐MALL outperformed BO and achieved a 10% improvement (141.84 MPa). Stagnation occurred after iteration 5, where the plateau coincided with a collapse in structural diversity, as samples clustered tightly in the design space (Figure 4f). It can be attributed to the reliance of its generative model on reconstruction loss, which prioritizes proximity to existing optima at the expense of exploration.

In contrast, MCTS‐AL delivered superior results, achieving considerably 20% increase in yield strength (154.09 MPa). While transient stagnation occurred between iterations 4 and 5 (likely due to CNN prediction errors), the framework maintained an upward trajectory, converging by iteration 6. MCTS‐AL's great improvement stemmed from two key features: diverse sampling and balanced exploration‐exploitation. The integration of the comprehensive performance criteria (see Experimental Section ‘Query Strategy’) with tree search generated structurally varied candidates while filtering poorly performing designs. Dynamic reinitialization of search trees prevented over‐reliance on historical optima and achieved a balance between exploration and exploitation, enabling sustained discovery of high‐performance regions. Scatter plots (Figure 4f) confirmed a more diverse sampling distribution of MCTS‐AL compared to GAD‐MALL and BO. By iteratively resetting search roots and prioritizing diverse candidates, MCTS‐AL avoided the local optima while efficiently converging to the global maxima, which is regarded as a critical advantage for solving high‐dimensional engineering problems like the design of mechanical metamaterials.

To characterize the structural diversity of optimized designs, we applied t‐Distributed Stochastic Neighbor Embedding (t‐SNE) to reduce the 27D design space to two dimensions (Figure 4f). For robust analysis, we also augmented the dataset with 2000 structures generated via Latin Hypercube Sampling (LHS, see Experimental Section ‘Structures Generation Strategy’). According to the results, BO and GAD‐MALL outputs remained largely indistinguishable from the initial dataset (Figure 4f), while MCTS‐AL generated designs with greater diversity and formed four distinct clusters, demonstrating its superior diversity in sampling strategy, which leads to more comprehensive understanding of the design space. Cluster 1 was positioned distally from the dataset center and exhibited the highest yield strength performance. Cluster 2 was marginally located with promising results, but was populated with statistically limited results (Only four valid results), and was excluded from the following analysis. Clusters 3 and 4 overlapped partially with the initial data, containing designs in those early iterations with moderate performances.

Focusing on clusters 1, 3, and 4, we observed increased diversity in volume fraction distributions compared to stiffness‐optimized designs (Figure S3, Supporting Information). While unit cells that shared *x*, *y* coordinates still maintained consistent trends, positional distributions diverged markedly. The central positions (e.g., (1, 1, δ_*z*_)) still have high volume fractions. However, three out of four positions (e.g., (0, 0, δ_*z*_), (2, 0, δ_*z*_), and (0, 2, δ_*z*_)) showed elevated volume fractions in strength‐optimized configurations. Besides, three side positions (e.g., (1, 2, δ_*z*_), (0, 1, δ_*z*_), and (2, 1, δ_*z*_)) exhibited low volume fractions, and the rest one ((1, 0, δ_*z*_)) displayed intermediate values. These findings suggest that high‐strength designs favor mass concentration in central and select corner regions. One thing needs to be mentioned is that structures with optimized specific stiffness also exhibited relatively high specific strength; meanwhile, structures with optimized specific strength also exhibited high specific stiffness. There are correlations but also differences between these two properties.

While MCTS‐AL outperformed GAD‐MALL and BO under fixed time constraints (6 h/iteration), each method has context‐dependent merits. BO is efficient for rapid latent space exploration but requires increased sampling density for high performance, escalating computational costs prohibitively. GAD‐MALL is effective for localized optimization but prone to stagnation due to limited generative diversity. By enforcing equal time budgets, we highlight MCTS‐AL's unique ability to balance global exploration with localized optimization, achieving superior efficiency and performance.

## 3 Conclusion

In this work, we present MCTS‐AL, a novel active learning framework that integrates CNN predictions, FEM simulations, and MCTS for the efficient optimization of 3D mechanical metamaterials. Compared to existing methods such as BO and GAD‐MALL, MCTS‐AL demonstrates faster convergence, superior optimization performance, and greater generalization capability. Using this framework, we successfully enhanced the specific stiffness and specific strength of Gyroid TPMS structures. Additionally, we thoroughly explored the relationship between spatial volume fraction distributions and the mechanical performance of the resulting designs. Key contributions and findings are summarized as follows:

- The framework requires only 100 initial samples to train CNN models to high accuracy, enabling efficient exploration of the design space with minimal data. Compared to BO and GAD‐MALL, MCTS‐AL converges more rapidly and yields structures with consistently better mechanical properties.
- Structures generated using MCTS‐AL achieved improvements of over 30% in specific stiffness and over 20% in specific strength, demonstrating the effectiveness of optimized volume fraction distributions.
- The observed correlations between volume percentage distributions and mechanical properties of the generated structures shed light on how structural geometry is refined by the AI‐based approach. Our results suggest that high‐performing TPMS metamaterials under uniaxial loading conditions benefit from greater alignment and consistency along the loading axis.

The MCTS‐AL framework balances exploration and exploitation by iteratively evolving a root structure and its offspring, each defined by a volume fraction matrix. This methodology is not restricted to TPMS structures and can be extended to other architected materials, especially those with non‐periodic or hierarchical features, where the primary challenge lies in multidimensional manufacturing and performance constraints, for instance, ensuring smooth transitions between adjacent building blocks. Future works may focus on integrating more advanced optimization algorithms to enhance working efficiency and expanding its application to higher‐dimensional design problems, thereby broadening its impact across materials science and engineering disciplines.

## 4 Experimental Section

### Structures Generation Strategy

Triply periodic minimal surfaces (TPMS) are implicit periodic surfaces defined by a single‐valued function in three‐dimensional space. These surfaces, represented as the zero set (or level set) of the function, partition space into two interpenetrating regions. In this work, we utilize the Gyroid (G) minimal surface, a type of TPMS defined by the equation:

(2) $$\emptyset_{G}\;\left(x,y,z\right)=\sin\left(X\right)\;\cos\left(Y\right)+\sin\left(Y\right)\cos\left(Z\right)+\sin\left(Z\right)\;\cos\left(X\right)=C$$

here, C controls the surface offset, which directly determines the volume fraction (and thus porosity) of the TPMS structure. By varying C, structures with distinct relative densities can be generated. The surface divides space into two phases (∅_*G*_ > *C* or ∅_*G*_ < *C*), with sheet or lattice TPMS structures differentiated by designating one phase as the void. We selected sheet‐type Gyroid structures due to their demonstrated mechanical superiority.

**Figure 5:**
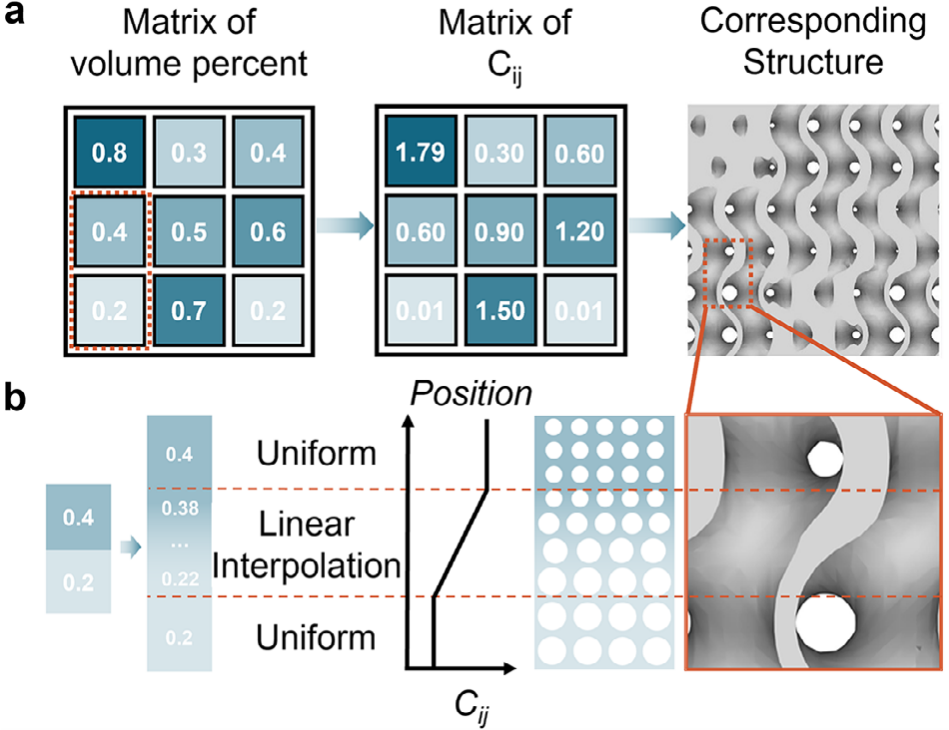
Structure generation strategy employed in MCTS‐AL. a) A volume percent matrix is first mapped to a corresponding matrix of material constants (C values), which is then used to generate the full 3D structure. b) Linear interpolation is applied to smooth the transition regions between different material phases, ensuring structural continuity and manufacturability.

To enable functionally graded designs, we replaced uniform porosity with a spatially varying volume percentage matrix, where the volume percentage is defined as the relative density (i.e., the volume of solid material divided by the total geometric volume of the sample). For instance, a structure measuring 9 mm × 9 mm × 9 mm with half its volume occupied by solid material has a volume percentage of 50%. To balance programmability and computational expense, each sample was designed with dimensions of 9 mm × 9 mm × 9 mm, discretized into a 3 × 3 × 3 grid comprising 27 unit cells. The volume fraction ρ of each cell was constrained to values between 0.2 and 0.8, incremented by 0.1. This range and resolution were selected to ensure design flexibility while maintaining manufacturability, as finer increments could exceed the practical accuracy limits of 3D printing and introduce mismatches between the designed and fabricated structures. Unlike uniform structures, our functionally graded metamaterials are characterized by spatially varying volume fractions across individual unit cells. These values were mapped to corresponding C parameters, forming a matrix *C_ij_* that defined graded structures. Linear interpolation between adjacent cells ensured smooth transitions, mitigating abrupt changes in boundary conditions (**Figure** **5**b). Post‐fabrication validation confirmed high accuracy, with actual volume fractions closely matching theoretical targets (Figure 5a). To maximize diversity in the small initial dataset (*n* = 100), we employed Latin Hypercube Sampling (LHS) to uniformly explore the design space. This approach ensured broad coverage of parameter combinations while maintaining statistical independence.

### Labeling with FEM Method

**Figure 6:**
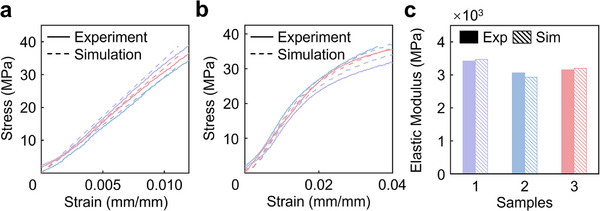
Comparison between FEM simulations and experimental results of the selected three samples. a) Experimental and simulation results for elastic modulus, where the solid line represents the average of experimental results over three replicates, and the dotted line corresponds to the simulation results. b) Experimental and simulation results for yield strength. c) Histogram comparing elastic modulus values of obtained from FEM simulations and physical experiments. The colors in Figure 6a,b are corresponding to the sample labels in Figure 6c, and the curves represent the mechanical response (e.g., stress–strain curves) of each of these three randomly selected samples. The experimental results closely match the simulations, with only minimal error, validating the accuracy of the simulation approach.

The mechanical properties of generated structures were evaluated via FEM simulations. Two distinct FEM models were implemented using the commercial software ABAQUS: Static 2019 for specific stiffness optimization and Explicit 2019 for specific strength optimization while using 16 CPU cores of an Intel Core i9‐13900K processor, without parallel processing. To validate simulation accuracy, compression tests were conducted on three representative TPMS structures, and results were compared against experimental data from 3D‐printed samples. Key parameters, including elastic modulus and plastic deformation curves, were calibrated to approximate the mechanical behavior of the printed materials (**Figure** **6**; Figure S4, Supporting Information). Three samples were selected randomly and simulated under compression to demonstrate the universal applicability and accuracy of our FEM configurations across diverse designs within the dataset. For each structure, three replicates per structure were tested to ensure reproducibility. The simulations exhibited strong agreement with experimental results (R > 0.95), yielding high‐quality datasets for surrogate model training.

### Monte Carlo Tree Search Active Learning Algorithm—Forward Prediction

CNNs were selected as surrogate models due to their ability to capture high‐dimensional spatial features in complex design spaces. In this work, the 27‐unit‐cell volume fraction matrix (3  ×  3  ×  3) served as input variables, with target outputs representing mechanical properties (e.g., elastic modulus, yield strength). Given the vast combinatorial design space (7^27^ configurations, derived from 7 discrete volume fraction levels per cell, as detailed in Experimental Section ‘Structures Generation Strategy’), CNNs provided an efficient framework for mapping structural configurations to performance metrics, which is a critical capability for navigating sparse, high‐dimensional optimization landscapes.

**Figure 7:**
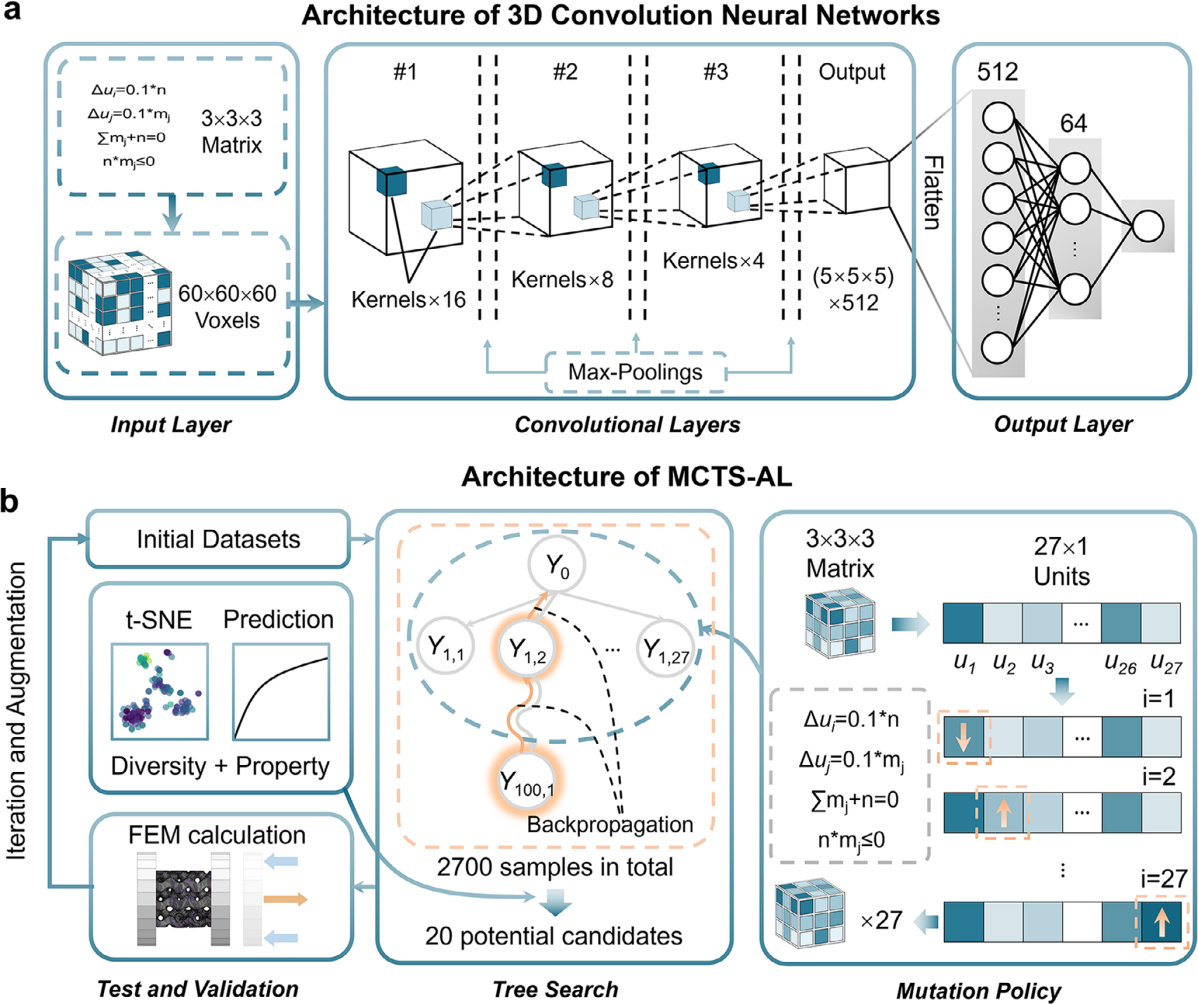
Network architecture and active learning strategy. a) Structure of the 3D CNN, consisting of an input layer, multiple convolutional layers, and an output layer for property prediction. b) Overview of the active learning framework. A total of 100 generations were sampled, each containing 27 structures. From each generation, 20 promising candidates were selected based on a combined evaluation of structural diversity and predicted performance.

The network architecture (**Figure** **7**a) comprised three core components: input layer, convolutional layers and fully connected layers. In the input layer, a 3  ×  3  ×  3 volume fraction matrix is voxelized to 60  ×  60  ×  60 resolutions to enhance feature extraction at material interfaces. The level‐set values (c) from the TPMS equation are first calculated based on the volume fraction matrix. An implicit surface was generated using the equation, and the design domain of 9 mm × 9 mm × 9 mm was discretized into a 60×60×60 grid. For each voxel, the left‐hand side of the TPMS equation (Equation 2 on page 15) was evaluated to determine whether it belonged to the solid (1) or void (0) region, thereby completing the voxelization. The dataset is split into a training and testing dataset with 80% and 20% proportions, respectively. The convolution layers consisted of three 3D convolutional layers that included a series of 3D convolution kernels extracting high‐dimensional information from the input structures. The first, second and third layers contained 16, 8, and 4 filters, respectively, and each of the layers was followed by a 3D max‐pooling layer responsible for down‐sampling. The pooling sizes were 3, 2, 2, which means, for example, the first layer shrinks the (60, 60, 60) boxes to (20, 20, 20) and takes the maximum of each (3, 3, 3) box as its output value. The last 3D convolutional layer was flattened into 512 neurons before reaching the output layers, followed by a fully connected layer with a size of 64 neurons. The activation function of 3D convolutional layers was the exponential linear unit (elu), and the loss function was the mean absolute error between the predictions and the test set. The training and inference of the CNN models were conducted on an Ubuntu system with an NVIDIA GeForce RTX 3060 GPU and without parallel processing.

### Monte Carlo Tree Search Active Learning Algorithm—Query Strategy

A MCTS method usually consists of two distinct policies: tree policy to select children and expand the tree, and default policy to produce a value estimate. In this work, we proposed an adapted MCTS method based on the scenarios of volume percentage matrix. The MCTS framework employs two complementary policies: a tree policy to select and expand nodes, and a default policy to evaluate candidate structures. In this work, MCTS was adapted to optimize volume fraction distributions in 3D mechanical metamaterials.

Unlike traditional MCTS methods, short backpropagation was incorporated into the MCTS‐AL framework, and the Upper Confidence Bound (UCB) formula was modified accordingly. The adapted UCB formula used in this work is defined as follows:

(3) $$UCB=v_{j}+0.95^{i}*c_{0}*\sqrt{\frac{lnN}{n_{j}+1}}$$

where, *v_j_* ​ denotes the performance prediction from the CNN model, *c*_0_ ​is the base exploration weight (set to 1000 for stiffness optimization and 100 for strength optimization), and* i* represents the current generation. Labeling with CNN predictions instead of FEM simulations significantly improves the efficiency of the workflow (Figure S9, Supporting Information). The term *N* corresponds to the visit count of the current root node, while *n_j_* refers to the visit count of its child node *j*.

In early optimization iterations, the exploration weight remains relatively high, promoting extensive exploration of the design space and enabling the selection of nodes even with moderately inferior performance. As iterations progress, the exploration weight decreases significantly, shifting the emphasis toward exploitation of the most promising regions identified. This adaptive balance between exploration and exploitation enhances the efficiency and effectiveness of the optimization process.

The tree policy is used for candidate generations. Each iteration begins by modifying the volume fraction matrix of the root structure. To maintain spatial coherence, only one unit cell is selected as the primary adjustment target, with its value perturbed within the allowable range (see Experimental Section ‘Structures Generation Strategy’). The perturbation scheme significantly influences the optimization outcome, as detailed in Figure S6 (Supporting Information). Specifically, as the number of unit cells perturbed per iteration increases, the optimization performance exhibits marked deterioration. This decline is attributed to the heightened stochasticity introduced by larger perturbations, which disrupts the balance between exploration and exploitation in the search process. The deviation from the original value is then distributed stochastically across the remaining cells, preserving the global volume fraction while ensuring exploratory diversity. This process generates 27 unique child structures per iteration, each corresponding to a distinct unit cell modification.

The default policy evaluates candidates' performances using pretrained CNNs, comparing predicted metrics between child structures and the current root node. After comprehensively weighing CNN predictions and visit counts (obtained via backpropagation) through the UCB formula, the optimized child structure is selected as the new root if it outperforms the current root node; otherwise, the current root is retained.

We also design random disturbance to ensure probabilistic retention of suboptimal candidates, preventing premature convergence to local maxima. This stochastic balancing enables robust global optimization, critical for navigating high‐dimensional design spaces.

### Monte Carlo Tree Search Active Learning Algorithm—Active Learning Workflow

The MCTS‐AL framework integrates CNN‐based forward prediction with MCTS‐guided exploration through three sequential phases: ensemble model training, candidate exploration, validation and iteration. First, five 3D‐CNN models were independently trained in a single optimization iteration on distinct 80:20 train‐test splits of the initial dataset to enhance prediction robustness and mitigate overfitting. Second, five root structures were selected (four top performers from the initial dataset and one randomly chosen candidate) to initiate the tree search process. Then each root underwent 100 MCTS iterations, generating ≤2700 candidates per root. All the candidates sampled in the tree search were ranked using a composite metric:

(4) $$\mathrm{Score}=\alpha *Y_{\mathrm{pred}}-\left(1-\alpha \right)*D_{\mathrm{design}-\mathrm{space}}$$

where Y_pred_ is predicted performance and D_design‐space_ quantifies dissimilarity from existing candidates. The top 20 candidates were selected to maximize diversity and performance. Finally, Selected candidates underwent FEM validation. Their results augmented the training set, and CNNs were retrained for subsequent generations.

The optimization process exhibits diminishing marginal returns, characterized by a substantial decline in the performance growth rate as iterations progress. Consequently, the workflow was terminated after three consecutive generations with <2% marginal improvement, where the result of each new iteration failed to exceed that of the current iteration by more than 2%. This termination criterion was decided by domain experience and trial‐and‐error procedures, representing a compromise between search depth and computational cost.

### Monte Carlo Tree Search Active Learning Algorithm—Additive Manufacturing by LPBF and Mechanical Properties Test

The structures were fabricated using additive manufacturing via Laser Powder Bed Fusion (LPBF) technology, employing WE43 magnesium alloy powder supplied by Tangshan Weihao Ltd. (Figure S3, Supporting Information). The specimens were fabricated using a BLT S210 metal 3D printer, configured with a laser power of 60 W, a scan speed of 800 mm s^−1^, and a layer thickness of 0.02 mm. Following printing, the samples underwent acid cleaning to remove sintered powder residues, ensuring the manufactured relative density accurately matched the designed volume fraction. Uniaxial compression tests were subsequently performed using an Instron testing system at a constant displacement rate of 1 mm/min, with strain measured directly using an extensometer. To focus on elastic modulus and yield strength characterization, the tests were terminated prior to specimen failure.

## Conflict of Interest

The authors declare no conflict of interest.

## Author Contributions

J.L. performed methodology, acquired software, and prepared the original draft. B.P. performed conceptualization and wrote‐ reviewing and editing. W.X. performed conceptualization, visualization, and wrote‐ reviewing and editing. Y.W. performed conceptualization and supervision. P.W. performed conceptualization, supervision, and acquired funding.

## Supporting information



Supporting Information

## Data Availability

The data that support the findings of this study are openly available in MCTS‐AL at https://github.com/Liujiamu24/MCTS‐AL, reference number 16261586.
