# Supplementary material for: Highly Efficient Discovery of 3D Mechanical Metamaterials via Monte Carlo Tree Search
Source: Adv Sci (Weinh). 2025 Sep 23;12(46):e13771. doi: 10.1002/advs.202513771 (PMC12697888; doi:10.1002/advs.202513771)
Supplement: Supplementary file 1 — Supporting Information [file ADVS-12-e13771-s001.docx]

Supporting Information

High-Efficient Discovery of 3D Mechanical Metamaterials via Monte Carlo Tree Search

Jiamu Liu^1^, Bo Peng^1^, Weiyun Xu^2*^, Ye Wei^3*^, Peng Wen^1*^

**Contents**

[S1. Specific Stiffness Optimization 3](#_Toc209101214)

[S2. Specific Strength Optimization 5](#_Toc209101215)

[S3. Characterization of WE43 Magnesium Alloy 6](#_Toc209101216)

[S4. Details in Finite Element Method (FEM) 7](#_Toc209101217)

[S5. Pseudocode of MCTS sampling 9](#_Toc209101218)

[S6. Sensitivity evaluation of the perturbation schemes of MCTS-AL 10](#_Toc209101219)

[S7. Robustness evaluation of extremely small dataset and noisy data 11](#_Toc209101220)

[S8. Structures optimization results of HEA 13](#_Toc209101221)

[S9. Efficiency comparison between FEM simulation and CNN prediction 16](#_Toc209101222)

S1. Specific Stiffness Optimization


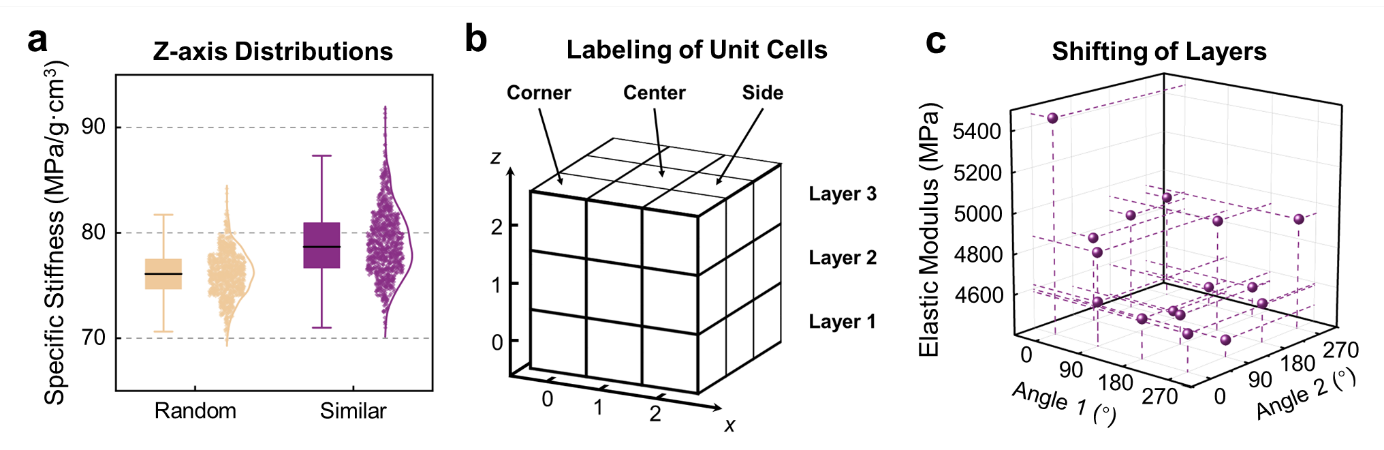


**Figure S1.** (a) 2000 graded Gyroid TPMS structures were generated, 1000 among which possessed random distributed volume percentage along the z axis while the remain half with similar distributions. (b) In order to better analyze the distribution of different unit cells, all of the unit cells are labeled with coordinates and are classified into 3 types: corners, centers and sides (c) A graded structure with similar volume percentage along z axis. 8 new structures were generated through shifting its second and third layers.


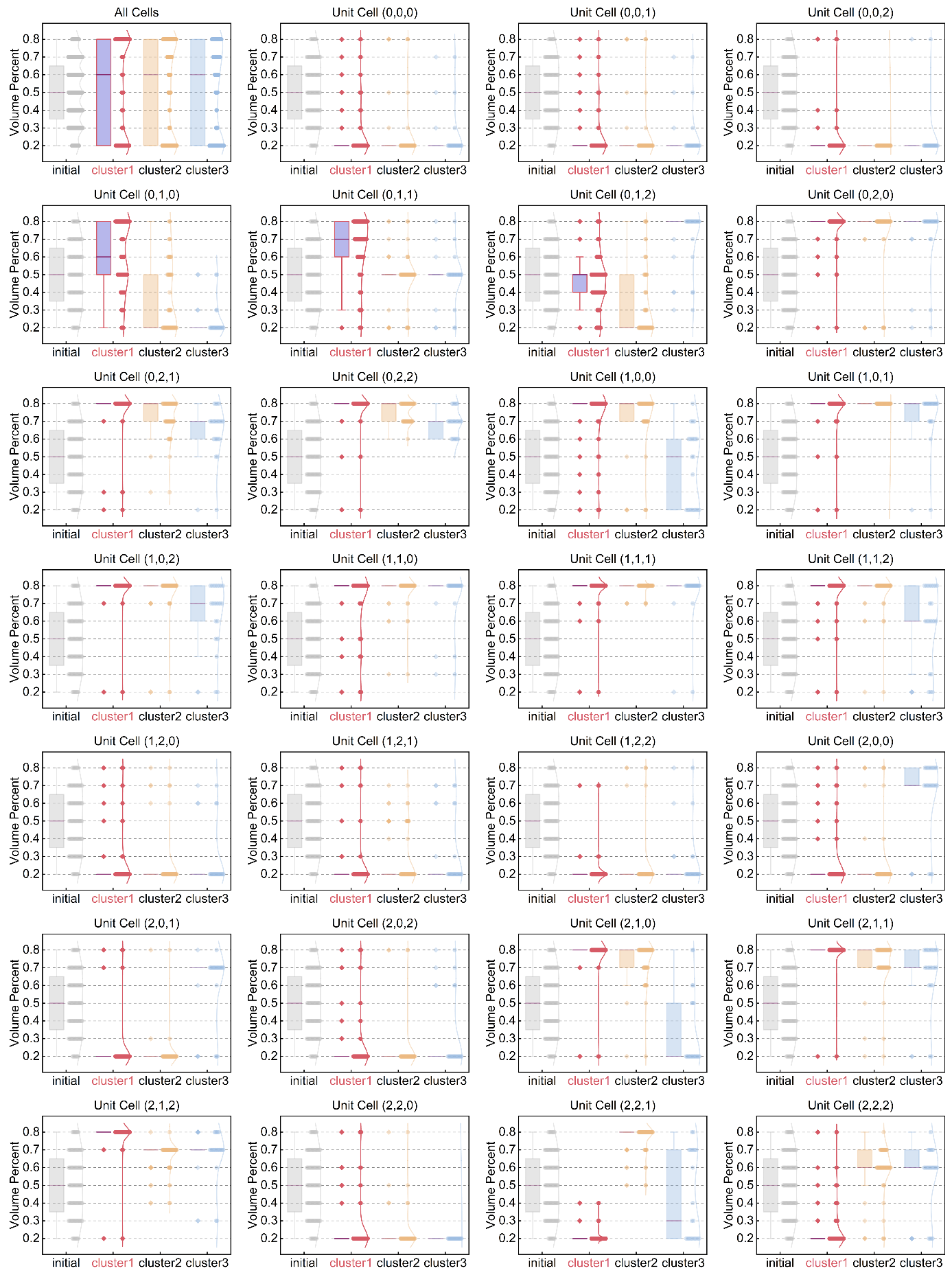


**Figure S2.** Unit cell distributions of different clusters in specific stiffness optimizations.

S2. Specific Strength Optimization


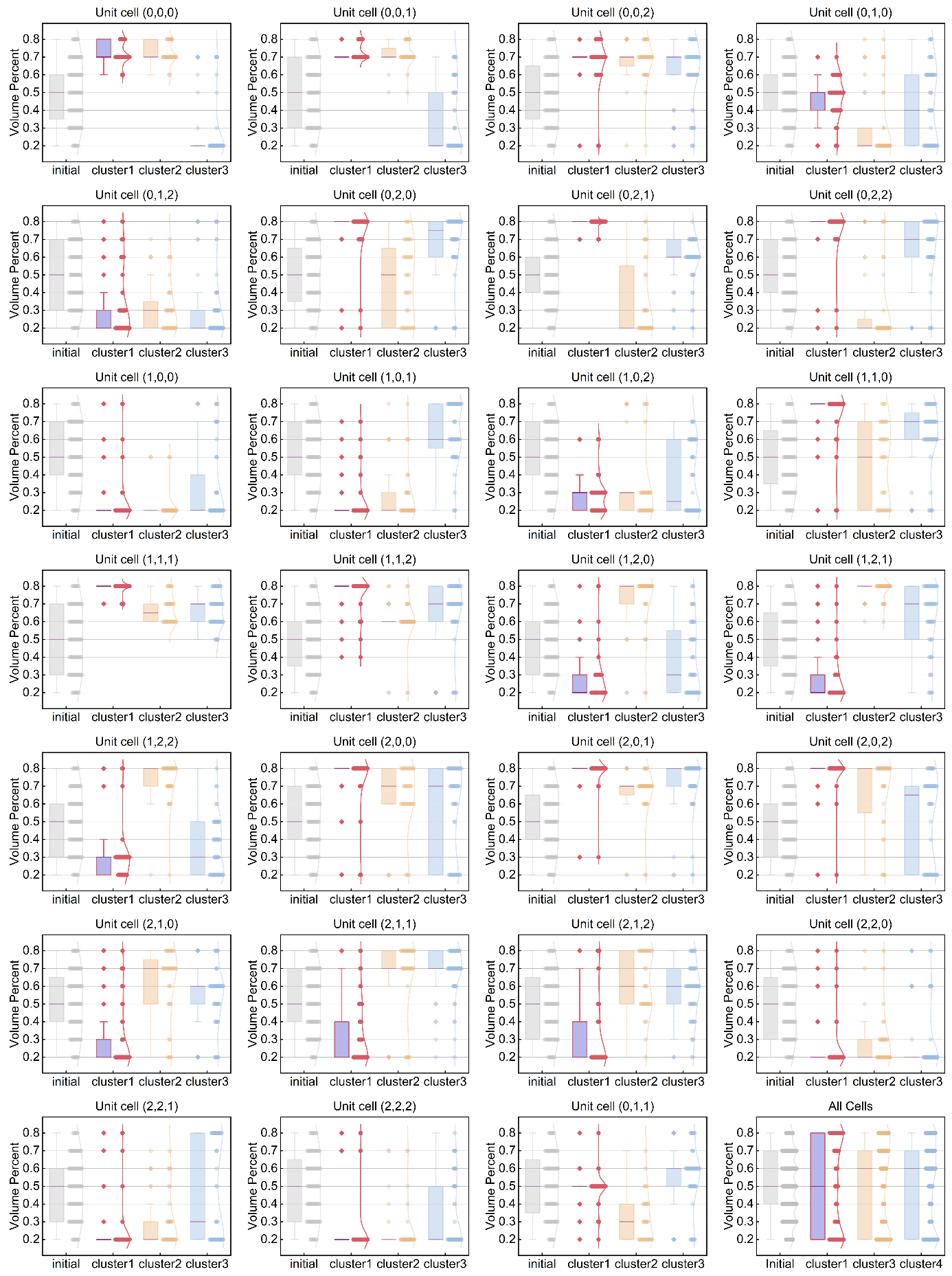


**Figure S3.** Unit cell distributions of different clusters in specific strength optimizations.

S3. Characterization of WE43 Magnesium Alloy


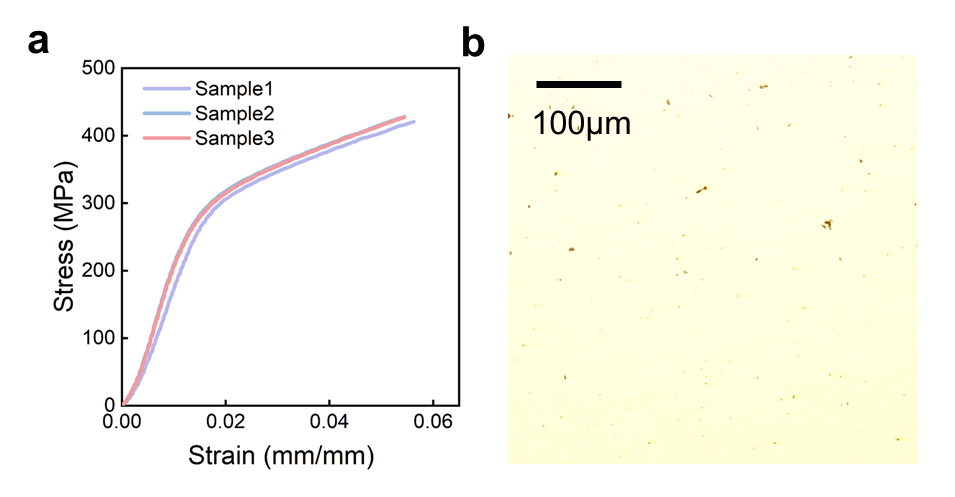


**Figure S4.** (a) Stress-Strain curves obtained through compress test of box samples printed by LPBF; (b) Section of box sample under optical microscope.

**Table S1.** Component of WE43 magnesium alloy powder though ICP.

| Element | Proportion(wt%) |
| --- | --- |
| Y | 3.38 |
| Nd | 1.95 |
| Gd | 1.12 |
| Zn | 0.19 |
| Zr | 0.33 |
| Mg | Bal. |

S4. Details in Finite Element Method (FEM)

Finite Element Method (FEM) simulations were performed according to the following procedure. First, HyperMesh (HM) was used to generate mesh models and corresponding .inp files. A mesh convergence study was conducted using element sizes of 0.2 mm, 0.12 mm, 0.08 mm and 0.06mm (results shown in Fig. S5). Based on a trade-off between computational expense and result accuracy, an element size of 0.08 mm was selected for all simulations. This decision reflects the substantially higher computational cost associated with finer meshes (models with an element size of 0.06 mm required several times the computational resources compared to the 0.08 mm configuration, but yields only a 1.7% improvement in accuracy). The files from HM were subsequently modified to define two node sets: a TOP set (nodes at the maximum z-coordinate) and a BOTTOM set (nodes at the minimum z-coordinate). The BOTTOM set was subjected to an ENCASTRE constraint, while a prescribed displacement was applied to the TOP set along the z-axis—0.1 mm for stiffness simulations and 0.6 mm for strength simulations. A linear elastic constitutive model was used for stiffness calculations, and an elastic-plastic model was employed for strength analysis. Following simulation, the effective stiffness was determined from the reaction force sum of the TOP node set and its average displacement. The material was modeled with a Young’s modulus of 12,000 MPa and a Poisson’s ratio of 0.34 for all simulations, with plastic behavior defined using the stress-plastic strain relationship provided in the subsequent table.


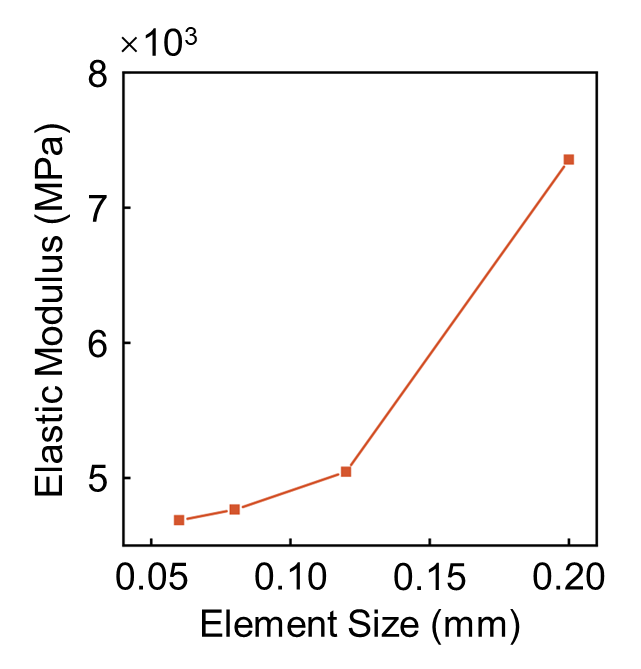


**Figure S5**. Reults of mesh convergence evaluation.

**Table S2.** Stress-plastic strain relationship

| Yield Stress (MPa) | Plastic Strain |
| --- | --- |
| 195.7  199.5  203.1  207.3  210.5  214.34  218.52  221.69  226.46  229.43  233.1  236.91  240.24  245.25  248.93  253.05  258.24  261.81  265.89  270.36  273.97  278.14  281.94  285.094  288.6  291.32  294.12  296.72  299.51  302.11  305.19 | 0  2.2E-4  2.6E-4  3.2E-4  3.7E-4  4.4E-4  5.3E-4  6E-4  7.2E-4  8E-4  9.2E-4  0.00105  0.00118  0.00141  0.00159  0.00182  0.00217  0.00244  0.00279  0.00324  0.00366  0.00422  0.00484  0.00543  0.00619  0.00687  0.00765  0.00842  0.00928  0.01004  0.01085 |

S5. Pseudocode of MCTS sampling

| Algorithm 1 Sampling through Monte Carlo Tree Search (MCTS) |
| --- |
| Input: Historical input of volume fraction matrixes {X_i_}, the modulus labels {y_i_} obtained by FEM simulations.  Output: 20 most promising candidates {$\hat{\mathbf{X}}$_i_}   1. N = dict[Any, int] // The visit counts of all the nodes explored 2. n_c_ = dict[Any, set] // The children nodes of a certain root node 3. v = dict[Any, float] // The prediction values of each node 4. r = 100 // number of max rollouts 5. Compute the average volume fraction and specific stiffness of {X_i_} 6. Select the initial root nodes {R_i_} from {X_i_} 7. N_all_ = [] // all the nodes explored in the sampling process 8. while i < r do 9. R_i_.findchildren(); //n_c_[R_i_] = [27 × n] 10. N[R_i_] += 1; 11. Select the n_max_ with highest UCT according to Eq.(3) 12. if UCT(R_i_) < UCT(n_max_) 13. R_i_ = n_max_ 14. end if 15. N_all_.append(n_c_[R_i_]) 16. end while 17. Select the candidates {$\hat{\mathbf{X}}$_i_} from N_all_ according to the criterion 18. return {$\hat{\mathbf{X}}$_i_} |

S6. Sensitivity evaluation of the perturbation schemes of MCTS-AL

Figure S6 compares the results of perturbing 1, 2, 3, 5, and 10 unit cells per iteration.


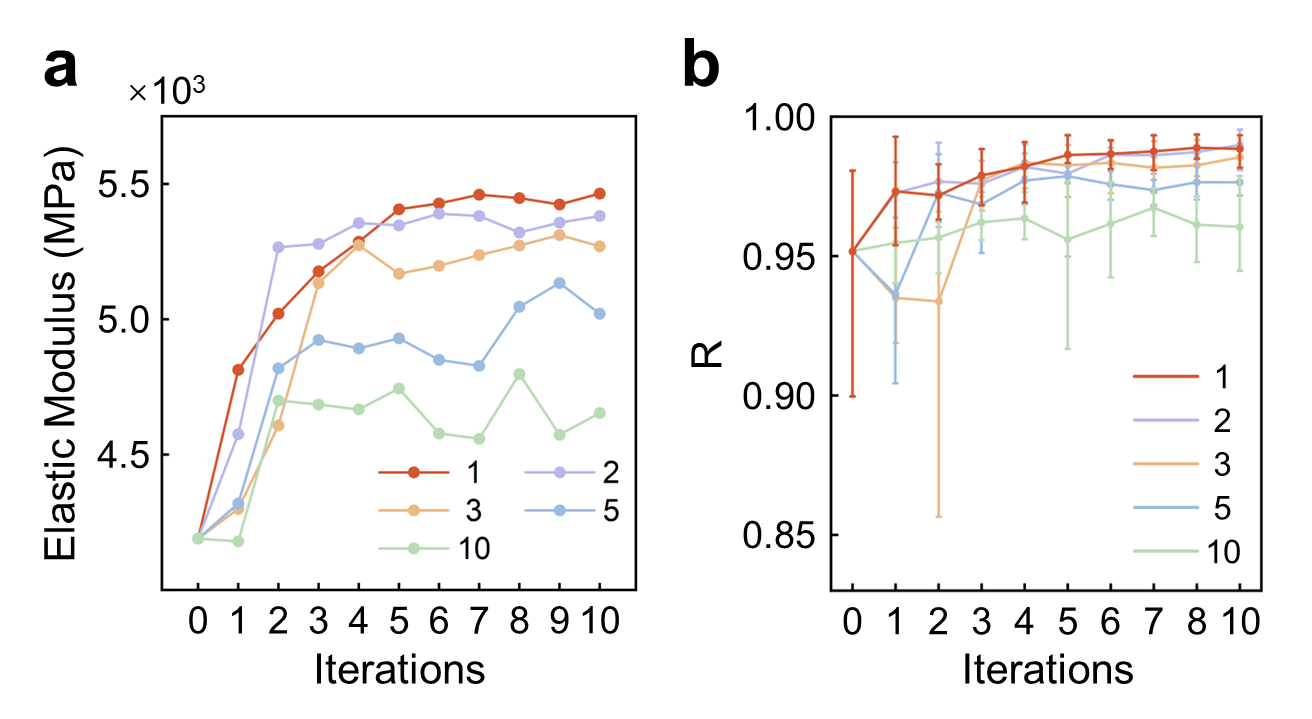


**Figure S6.** Optimization performances with different perturbation schemes. (a) Highest elastic modulus in the candidates of each iteration. (b) Correlation efficient (R) of the CNN models in each iteration.

The results indicate that the perturbation scheme considerably affects the performance of MCTS-AL. Specifically, as the number of perturbed cells increases, the final elastic modulus exhibits a marked decline. To ascertain that this trend was not due to prediction inaccuracies, we examined the model's performance throughout the optimization. As depicted in Figure S6b, all groups achieved high model accuracy, with correlation coefficients (R) between 0.95 and 0.97, indicating that prediction error was not a contributing factor. Instead, the variation in outcomes is attributable to differences in mutation strategies. While MCTS balances exploration and exploitation, perturbing more cells introduces greater stochasticity into the exploration process. This heightened randomness ultimately renders the search functionally analogous to stochastic sampling around top-performing designs—a behavior consistent with the principles underlying GAD-MALL.

S7. Robustness evaluation of extremely small dataset and noisy data

Specific stiffness optimization task using MCTS-AL with initial dataset sizing 20 and 60 were conducted to evaluate the robustness of extremely small dataset. As shown in Fig. S7a, the optimization process starting with only 20 samples exhibited high stochasticity in its initial stages and exhibited no measurable improvement until the 7th iteration, where there were already 160 samples available for training the CNN models. In contrast, the process starting with 60 initial samples demonstrated more stable and superior performance, with elastic modulus of selected candidates increasing steadily since the 3rd iteration.

Despite all groups expanding their datasets to 100 samples in subsequent iterations, the three trials (including 100 initial samples) concluded with markedly different results after 10 iterations. This divergence is likely attributable to differences in how effectively the initial samples characterized the design space. The samples augmented based on CNN predictions were less effective at capturing the full design space than those initial ones generated by Latin Hypercube Sampling (LHS). Consequently, the ML workflow requires more data to achieve an optimization performance equivalent to that of a well-designed initial sample set. In a conclusion, we found that 100 initial samples were the reasonable minimum dataset.


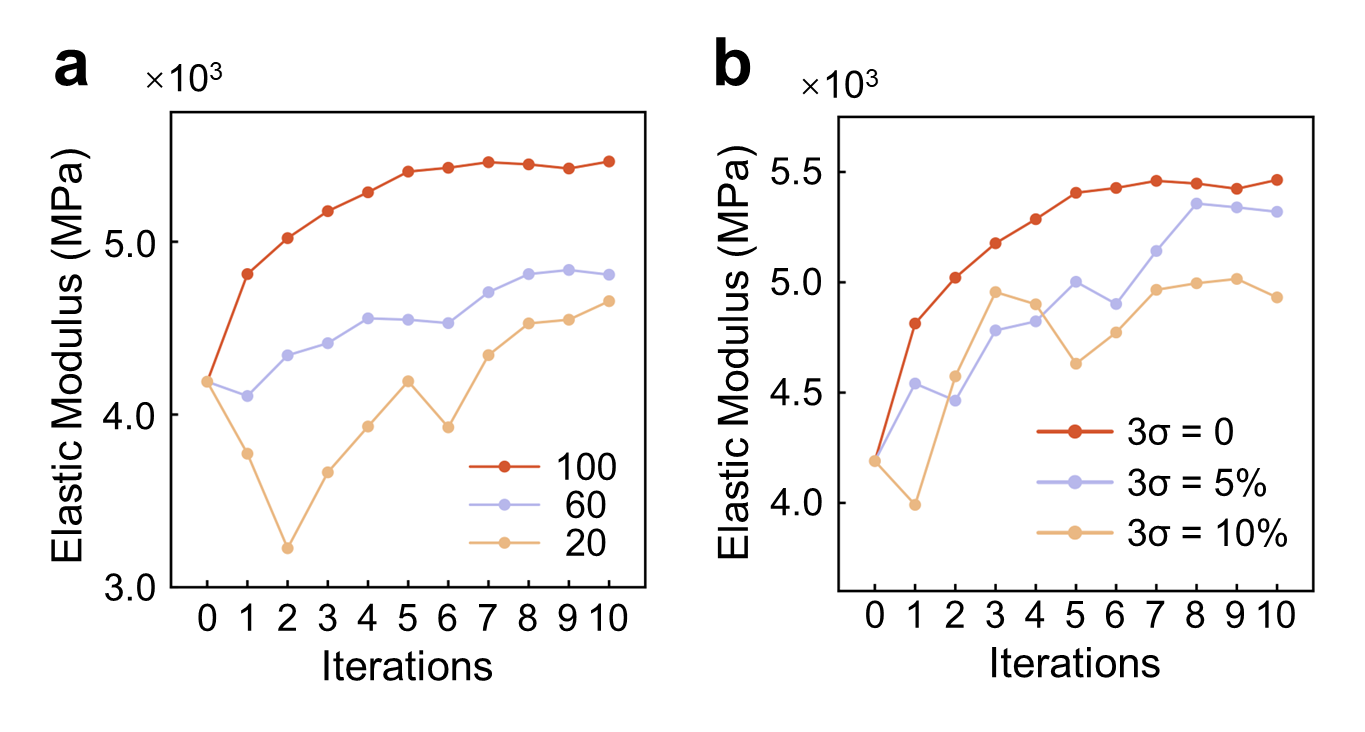


**Figure S7**. Optimization performances of initial datasets with different sizes, and data with noise of different levels. (a) Optimization performances of different initial datasets, sizing 100, 60 and 20 respectively. (b) Optimization results when there are noisy data or incorrect data. Data labels in group 2 and 3 were added a Gaussian noise.

Gaussian noise (mean = 0) was introduced to the labels of all samples acquired during the optimization process to evaluate the robustness against inaccurately labeled or noisy data. The noise level was controlled such that three standard deviations (3σ) corresponded to 5% and 10% of the mean performance value of the initial dataset.

As shown in Fig. S7b, the presence of noise adversely affected the optimization outcomes and hindered the algorithm's capacity to identify optimal designs. The performance degradation was more pronounced with higher levels of noise. Despite this, the method consistently generated structures exhibiting stiffness enhancements greater than 20%. This resilience is likely attributable to the comprehensive selection criterion of MCTS-AL, which incorporates both predicted performance and sample diversity. This dual strategy of balancing exploration and exploitation appears to be instrumental in maintaining robustness when processing noisy data.

S8. Structures optimization results of HEA

The architecture of HEA algorithm applied in this article is illustrated in Fig. S8. The fitness *F* of each individual is calculated using the following expression:

$$F=v_{i}+{0.95}^{A}*c_{0}*\sqrt{\frac{lnA}{a_{j}+1}} (5)$$

where *v_j_*​ is the performance predicted by the CNN model, and *c*_0​_ is a base exploration weight set to 1000 for stiffness optimization and 100 for strength optimization. Here, *A* denotes the total number of rounds elapsed since the evolution began, and *a_j_*​ represents the age of individual *j*, which increases by 1 if the individual is retained after the population update in a round.

The algorithm initializes by evaluating the fitness of the initial population and selecting the main population based on their fitness. Each year, 50 hybridization events occur: parents are selected with probabilities $P_{ci}$ proportional to their fitness, given by:

$$P_{ci}=\frac{F_{i}}{\sum F_{j}} (6)$$

where $F_{i}$ is the fitness of individual *i* and the denominator is the sum of all fitness values in the population. The offspring is generated as a convex combination of the two parents. Mutation occurs with a 50% probability, in which one randomly chosen position of the offspring is regenerated. The fitness of each new offspring is evaluated through the CNN model. Subsequently, low-fitness individuals are eliminated to maintain a stable population size.

The evolution process spans 500 rounds. After completion, all individuals generated over the entire evolutionary history are recorded, and 20 promising candidates are selected according to their scores *v_j_*​.


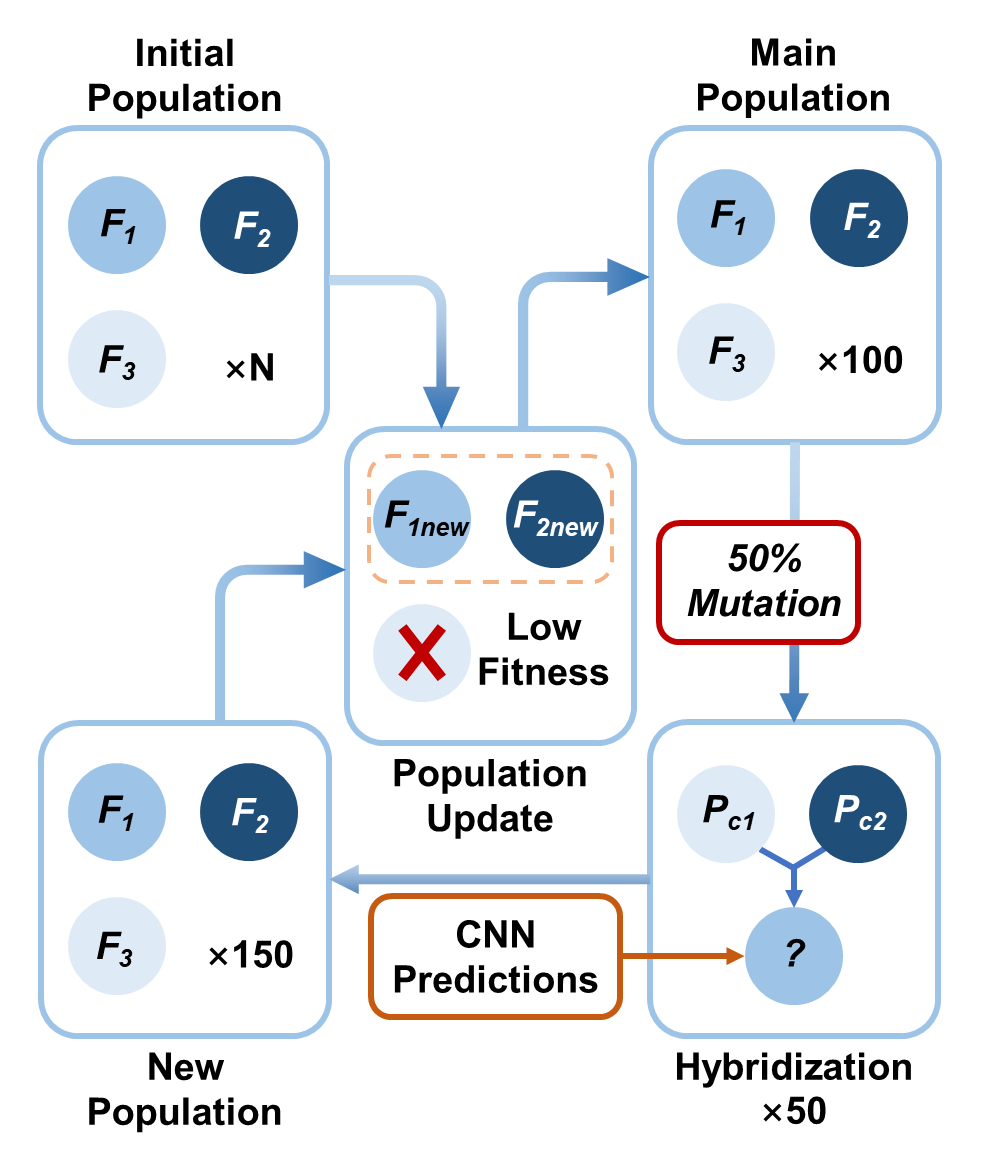


**Figure S8**. Structure of HEA integrating CNN predictions and Upper Confidence Bound (UCB).

The optimization results for both specific stiffness and strength are presented in Fig. S9. Both optimization processes exhibit similar trends: rapid initial improvement, followed by diminishing returns in later stages. This pattern may be attributed to characteristics of the hybridization process: Structures with symmetrical designs often exhibit similar mechanical properties, and hybridizing two symmetrical parent structures tends to produce offspring with reduced geometric variation. Since mechanical performance in metamaterials often benefits from controlled non-uniformity—where strategic gradients enhance properties—this increased uniformity may limit further performance gains.

Additionally, although the mutation rate was set as high as 50%, the HEA workflow still demonstrated limited exploratory capacity. This resulted in premature convergence and significant clustering among the final candidate structures, further restricting diversity and hindering the discovery of superior designs.


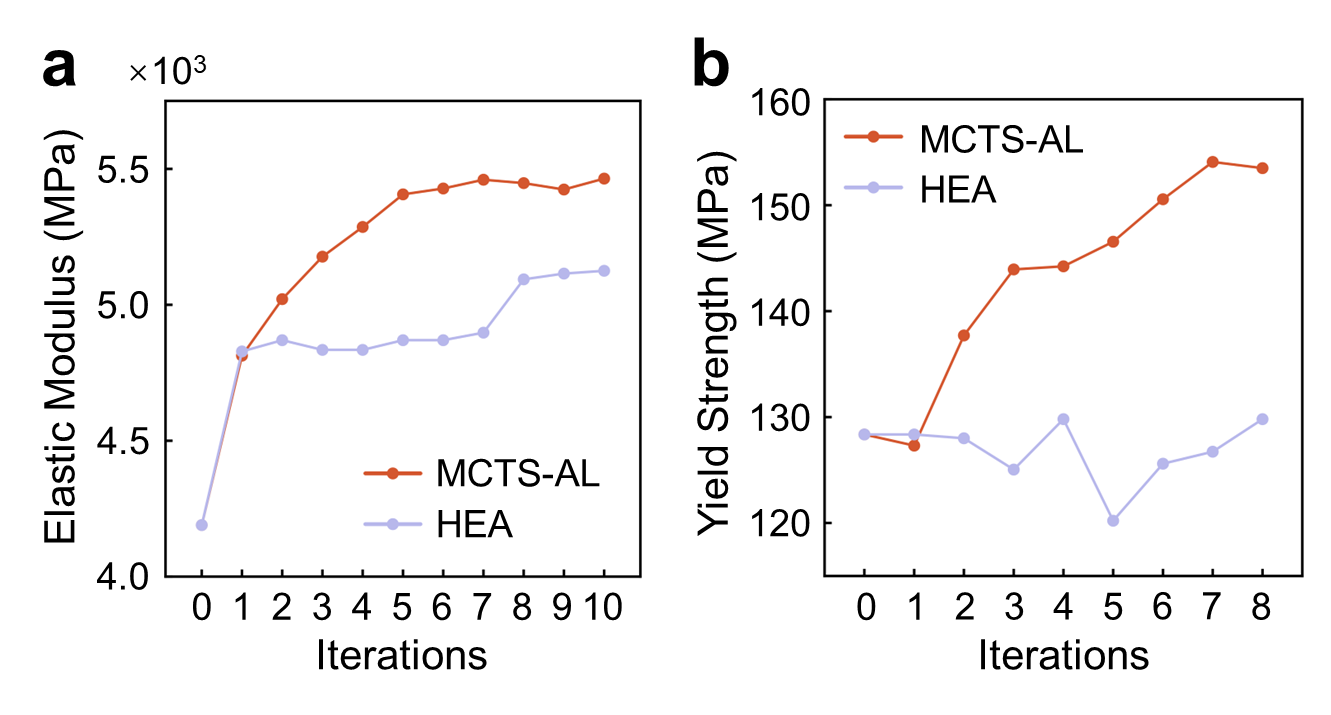


**Figure S9**. Optimization results of HEA applied in the specific stiffness (a) and strength (b) tasks.

S9. Efficiency comparison between FEM simulation and CNN prediction

To more intuitively compare the efficiency of finite element method (FEM) simulations and convolutional neural network (CNN) predictions, the time required to process 100 samples for stiffness labeling for both methods were evaluated. The configurations for simulating stiffness and strength differ significantly: stiffness simulations use a linear elastic constitutive model, while strength simulations employ an elastic-plastic model, which is computationally more expensive. Neither of the CNN or FEM method utilized parallel processing during these comparisons.


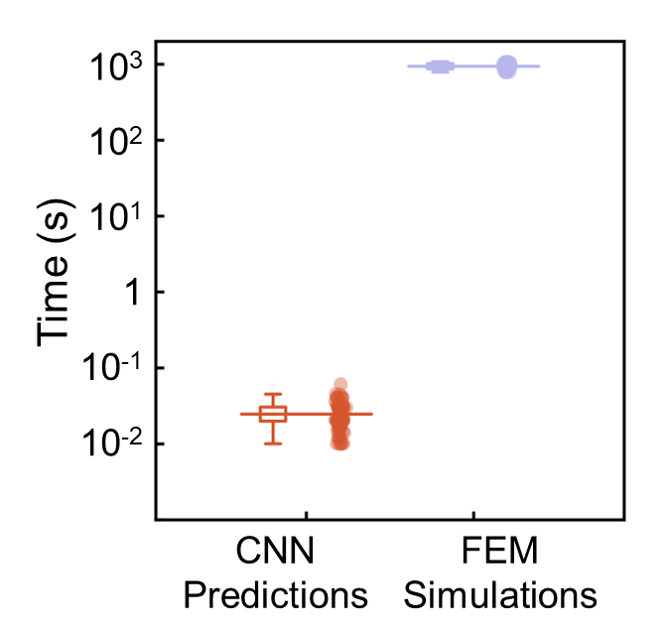


**Figure S10.** Time consumption for labeling 100 randomly selected samples with stiffness.

As shown in Fig. S10, obtaining mechanical properties through FEM simulations is orders of magnitude more time-consuming than using CNN predictions. This computational gap will be particularly pronounced for complex properties such as yield strength, where FEM requires significantly more resources, further widening the disparity. Consequently, CNN-based predictions serve an essential role in efficiently generating labels during the Monte Carlo Tree Search with Active Learning (MCTS-AL) sampling process.
